# Cytoplasmic Continuity Revisited: Closure of Septa of the Filamentous Fungus *Schizophyllum commune* in Response to Environmental Conditions

**DOI:** 10.1371/journal.pone.0005977

**Published:** 2009-06-22

**Authors:** Arend F. van Peer, Wally H. Müller, Teun Boekhout, Luis G. Lugones, Han A. B. Wösten

**Affiliations:** 1 Department of Microbiology, Institute of Biomembranes, Utrecht University, Utrecht, The Netherlands; 2 Department of Cellular Architecture and Dynamics, Institute of Biomembranes, Utrecht University, Utrecht, The Netherlands; 3 CBS Fungal Biodiversity Centre, Utrecht, The Netherlands; University of Missouri-Kansas City, United States of America

## Abstract

**Background:**

Mycelia of higher fungi consist of interconnected hyphae that are compartmentalized by septa. These septa contain large pores that allow streaming of cytoplasm and even organelles. The cytoplasm of such mycelia is therefore considered to be continuous.

**Methodology/Principal Findings:**

Here, we show by laser dissection that septa of *Schizophyllum commune* can be closed depending on the environmental conditions. The most apical septum of growing hyphae was open when this basidiomycete was grown in minimal medium with glucose as a carbon source. In contrast, the second and the third septum were closed in more than 50% and 90% of the cases, respectively. Interestingly, only 24 and 37% of these septa were closed when hyphae were growing in the absence of glucose. Whether a septum was open or closed also depended on physical conditions of the environment or the presence of toxic agents. The first septum closed when hyphae were exposed to high temperature, to hypertonic conditions, or to the antibiotic nourseothricin. In the case of high temperature, septa opened again when the mycelium was placed back to the normal growth temperature.

**Conclusions/Significance:**

Taken together, it is concluded that the septal pores of *S. commune* are dynamic structures that open or close depending on the environmental conditions. Our findings imply that the cytoplasm in the mycelium of a higher fungus is not continuous *perse*.

## Introduction

A fungal mycelium is the result of fusing hyphae that grow at their apices and that branch subapically. In general, hyphae of the lower fungi, i.e. the Glomeromycota, Zygomycota, and Chytridiomycota are sparsely, if at all, septated [1]–[3]. Therefore, the cytoplasm within mycelia of these fungi is continuous. Hyphae of the higher fungi, i.e. the Ascomycota and Basidiomycota, are compartmentalized by septa. These septa contain central pores of up to 500 nm that allow streaming of cytoplasm and translocation of organelles like mitochondria and nuclei [1]–[3]. Therefore, the cytoplasm within these mycelia is also considered to be continuous. This discriminates the filamentous fungi from plants and animals. In these latter two kingdoms there are also intercellular cytoplasmic connections but they are much smaller. Gap junctions in animals and plasmodesmata in plants have pores with a diameter of about 1.5 to 3.0 nm. These pores allow streaming of inorganic ions and small water-soluble organic molecules [8]–[10]. It should be noted that the diameter of the pores of plasmodesmata and gap junctions is dynamic. For instance, the channels in plasmodesmata can be closed or their width increased to 5 to 9 nm.

The major groups of fungi within the Basidiomycota contain different types of septa. The Pucciniomycotina and the Ustilaginomycotina have relatively simple septa [1], [11], [12]. In contrast, septa of the Agaricomycotina are relatively complex. They consist of a barrel-shaped swelling around the pore, the dolipore, which is associated with a septal pore cap (SPC) [6]. This septal pore cap, which restricts organelle translocation, can be of the vesiculate type, the perforate type or the imperforate type [13] and is assumed to be derived from the endoplasmic reticulum [14]–[16]. The SPC of *Schizophyllum commune* is of the perforate type. Its base, i.e. the part closest to the septum, has a diameter of 450–600 nm and the whole structure is regularly perforated by openings of approximately 100 nm [15], [17].

Septa of Ascomycota and the Basidiomycetes become plugged in response to hyphal damage to prevent loss of cytoplasm [18]–[20]. Peroxisome-like organelles, called Woronin bodies, plug the septa of the ascomycetes [21]–[23], whereas in basidiomycetes septa are closed by electron dense, plugging material [19]. It has been proposed that the SPC is involved in the plugging process [15], [20], [24], [25]. Here, it is shown by laser dissection that septa of growing hyphae of *S. commune* not only plug in response to hyphal damage but that this is also caused by environmental conditions such as availability of carbon source, exposure to high temperature, osmotic shock or toxic agents. The results thus imply that the cytoplasmic continuity of this fungus depends on the environmental conditions.

## Results and Discussion

**Figure 1:**
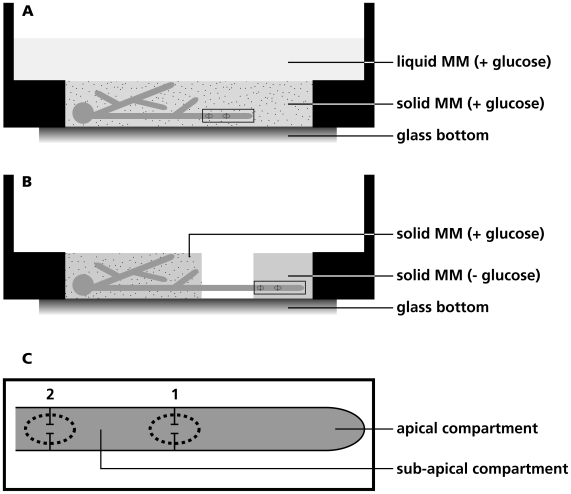
Glass bottom culture dish used to assess plugging of septa in *S. commune*. In most cases *S.commune* was grown in a thin layer of agar medium overlaid with minimal medium (A). However, liquid medium was not added when it was assessed whether septa were open or closed in the absence of glucose in the medium. In this case, the culture dish contained distinct patches of minimal medium with or without glucose, which were separated by a gap of 5 mm (B). (C) Magnification of boxed area in (A) and (B) showing a hypha with the first and second septum as referred to in the text.

**Figure 2:**
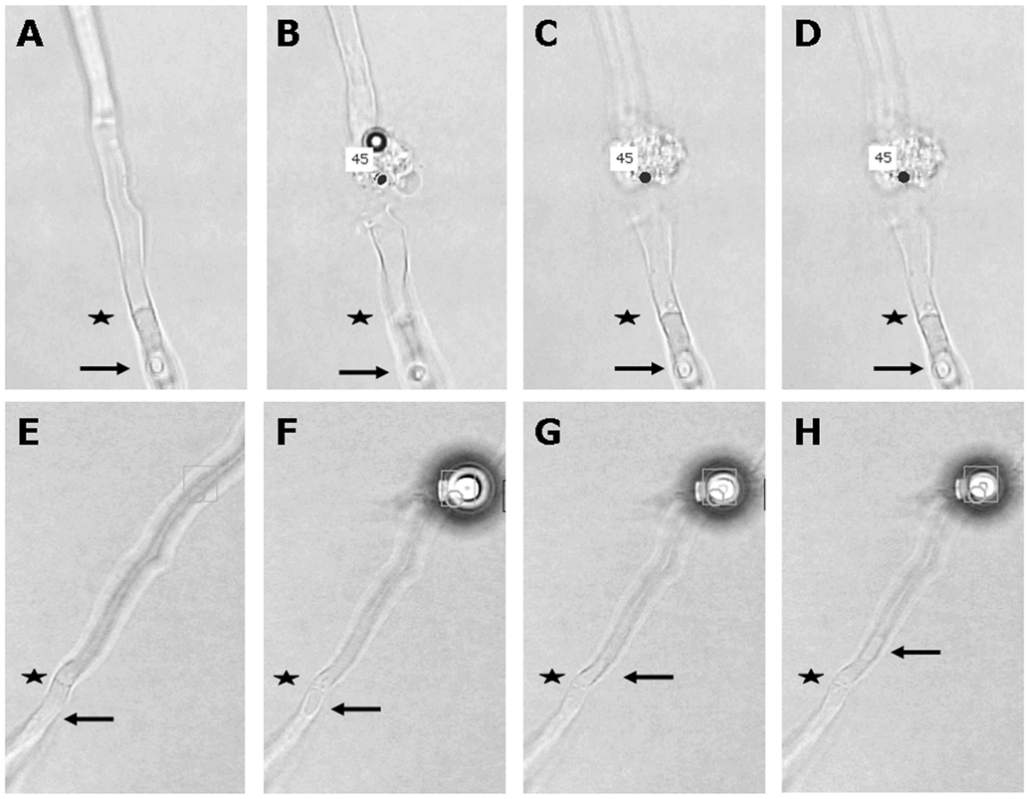
A septum is open when cytoplasm moves through the septum after laser dissection of a neighbouring compartment. Compartments before (A, E) and after (B–D, F–H) dissection of a hypha with a closed (A–D) and an open (E–H) septum. Cytoplasmic flow in E–H is indicated by the movement of a vacuole (arrows) through the septum (*). Dots represent the position of the laser.

*S. commune* was grown in a glass bottom culture dish in a thin layer of solidified minimal medium (MM) containing glucose as a carbon source. The solidified medium was overlaid with liquid MM (Figure 1A). Extension of selected hyphae was followed during a 3 min period using the light microscope of the PALM CombiSystem. Compartments of growing hyphae were disrupted with the laser of the PALM Combi-system within 30 µm from the septum. Cytoplasm of disrupted compartments spilled into the surrounding medium (Figure 2). Cytoplasmic flow from the adjacent compartment into the medium depended on the state of the septum (Figure 2; Movies S1, S2, S3, S4, S5, S6). Loss of cytoplasm was considerable (septum was open and was slowly closed; Movies S5-S6), minor (septum was open but was quickly closed; Movies S3-S4) or not detected (septum was already closed; Movies S1-S2). By cutting the second compartment and following cytoplasmic streaming from the apical compartment into the medium it was shown that 45 out of 45 apical septa were open (Table 1). In contrast, only 25 out of 53 of the second septum (separating compartment 2 and 3; see Figure 1C) and 1 out of 14 of the third septum (separating compartment 3 and 4) were open. This was shown to occur in growing hyphae throughout the mycelium. The second and third septa closed quickly in the case they were open. In contrast, closure of the most apical septum was generally slower.

**Table 1:** Plugging of a septal pore depends on its position in the hypha and the presence of glucose in the medium.

Treatment	Septum*	Total number of Septa	Open Septa	Closed Septa	% Open Septa
MM + glucose	1	45	45	0	100
	2	53	25	28	47
	3	14	1	13	7
MM - glucose	1	12	11	1	92
	2	29	22	7	76
	3	41	26	15	63

* Septum 1 separates the apical compartment from the second compartment; septum 2 separates compartment 2 and 3, and septum 3 compartment 3 and 4.

The fact that the second and third septa were often closed came to a surprise considering the phenomenon of streaming of cytosol in a fungal mycelium [26]. We argued that in the presence of a surplus of glucose in the medium cytosolic streaming is not essential and that therefore septal pores can be closed. To test this hypothesis, hyphae were grown from a glucose-containing medium into a medium without this carbon source (Figure 1B). Indeed, in MM without glucose many more septa of growing hyphae were open (i.e. 11 out of 12 apical septa, 22 out of 29 second septa and 26 out of 41 third septa; Table 1). Taken together, these findings show that the continuity of the cytoplasm of *S. commune* depends on the presence of carbon source in the medium.

We reasoned that another environmental condition that could affect the septal pore is exposure to stress. To test this, the liquid medium overlaying the solid minimal medium (Figure 1A) was replaced by deionized water. This neither affected growth nor closure of the apical septum in a 60 minutes interval (Table 2). In contrast, addition of 1 M MgSO_4_ to the liquid medium did have an effect on the state of the septal pore. Addition of MgSO_4_ initially resulted in the accumulation of vacuoles and after 15 minutes most hyphae had stopped growing. At this point, all apical septa were still open. However, 5 out of 5 apical septa had closed after an additional 30 minutes of exposure to 1M MgSO_4_. Absence of streaming of cytoplasm into the medium from the adjacent compartment was not due to the presence of vacuoles near the septum. Exposure of *S. commune* to 20 µg ml^−1^ nourseothricin, which inhibits protein synthesis, triggered a similar response as 1M MgSO_4_ (Table 2). Within 30 minutes, the hyphal tips stopped growing and mild vacuolization was observed. Yet, septa were still open. After another 30 minutes, all hyphae were heavily vacuolized and almost all apical septa had closed. Transfer of the mycelium from 25°C to 0 or −20°C did not cause plugging. Hyphae continued their original growth rate when they were placed back at 25°C. Exposing the mycelium to 45°C for 30 minutes stopped growth. Hyphae vacuolized and all apical septa had closed (Table 2). Interestingly, most septa opened again 15 minutes after colonies were placed back at 25°C. During this time, hyphae restored normal growth and vacuolization was decreased to normal levels.

**Table 2:** Plugging of the apical septum depends on environmental stress.

Treatment*	Total number of Septa**	Open Septa	Closed Septa	% Open Septa
Hypotonic, 45 min	5	5	0	100
Hypertonic, 45 min	5	0	5	0
20 µg ml^−1^ nourseothricin	5	1	4	20
45°C, 30 min	5	0	5	0
45°C, 30 min; 25°C, 15 min	5	4	1	80
0°C, 30 min	5	4	1	80
−20°C, 30 min	5	5	0	100

* Hyphae were grown in MM medium with glucose. Hypotonic or hypertonic conditions were created by overlaying the agar medium with water and 1 M MgSO_4_, respectively. Nourseothricin was added to the liquid medium overlaying the agar medium.

** Hyphae were analysed in two independent experiments.

Our data show that hyphae initially accumulate vacuoles upon exposure to stress, after which their growth halts. This is followed by septal closure. Closure of the septum may thus be a final rescue system that is activated to have individual compartments survive the stress condition. Notably, plugging was shown to be reversible, at least when induced by heat. This indicates that septal pores are dynamic structures that can open and close depending on the environmental conditions. Taken together, it is concluded that the cytoplasm of a mycelium of a higher fungus is not continuous *per se*, as is generally assumed. The cytoplasmic connections within a mycelium of *S. commune* thus resemble those in plants and animals. Like the septal pore of this basidiomycete, plasmodesmata and gap junctions can reversibly open and shut [8], [27], [28].

## Materials and Methods

### Strains and growth conditions

*S. commune* strain 4–8 (FGSC # 9210 VT # H4–8) was grown in the light at 25°C on minimal medium with 2% glucose (MM; 29). Plugging experiments were performed in glass bottom culture dishes (P35G-0-20-C, MatTek Corporation, Ashland, MA, USA). To this end, wells in the dishes (20 mm in diameter, 1 mm in height) were filled with 400 µl MM containing 1% agarose. Cultures were inoculated with a plug of *S. commune* mycelium that was gently pushed in the agar medium containing glucose. Dishes were filled with 2 ml liquid MM (Figure 1A) and transferred to a water vapour saturated chamber at 25°C for 2–3 days. Heat stress was applied by floating the glass bottom culture dish in a water bath at 45°C. Alternatively, wells were filled with MM that contained glucose on one side but no carbon source on the other side (Figure 1B). These media were separated by a 5 mm gap preventing glucose to diffuse into the medium without carbon source. In this case the agar media were not topped with liquid medium after inoculation with a plug of mycelium.

### Analysis of plugging

Glass bottom culture dishes were mounted on a PALM CombiSystem (Carl Zeiss MicroImaging GmbH, Munich, Germany). Disruption of compartments was performed with laser pulses (laser setting “dots”, laser power 65%). Movies were captured to assess whether septa were open or closed. To this end, spilling of cytoplasm from compartments adjacent to the disrupted compartment was monitored.

## Supporting Information

Movie S1 This septum is closed. There is no spilling of cytoplasm from the compartment that is adjacent to the one that is damaged.
(6.14 MB MP3)

Movie S2 This septum is closed. There is no spilling of cytoplasm from the compartment that is adjacent to the one that is damaged.
(2.40 MB MP3)

Movie S3 This septum is open but it closes quickly after the compartment is damaged. There is minor spilling of cytoplasm from the compartment that is adjacent to the one that is damaged.
(6.65 MB MP3)

Movie S4 This septum is open but it closes quickly after the compartment is damaged. There is minor spilling of cytoplasm from the compartment that is adjacent to the one that is damaged.
(3.62 MB MP3)

Movie S5 This septum is open and it closes slowly after the compartment is damaged. There is major spilling of cytoplasm from the compartment that is adjacent to the one that is damaged.
(1.80 MB MP3)

Movie S6 This septum is open and it closes slowly after the compartment is damaged. There is major spilling of cytoplasm from the compartment that is adjacent to the one that is damaged.
(0.15 MB MP3)
